# To Shorten and Lessen the Pain of Natural Labor

**Published:** 1884-06-20

**Authors:** John Morris

**Affiliations:** Baltimore


### WHAT MEANS CAN BE JUDICIOUSLY EMPLOYED TO SHORTFN THE TERM AND LESSEN THE PAIN OF NATURAL LABOR?

BY JOHN MORRIS, M. D., OF BALTIMORE.

**Read before the Section on Obstetrics and Diseases of Women, June, 1883.**

In no one thing has the wisdom and genius of the age been more thoroughly exhibited than in the advance and elevation of the art of midwifery. Once considered an inferior branch of medicine, it has, through the vigor and enlightenment of those pursuing it, risen to the highest rank in the scale of the sciences. Men have learned to properly appreciate the knowledge and skill by which, in the most critical hours of existence, pain is ameliorated, sorrow assuaged, and life saved. Through the goodness of God, the original curse placed on the mothers of men has been softened, if not nullified. "In sorrow thou shalt bring forth children," will, before this generation has passed away, be but a record of the agony of the past.

In the consideration of this subject, my purpose is to present some views to you, based on my own practice, concerning the management of women in labor. My object in the presentation of these views is to elicit discussion and to bring out the experience of the gentlemen assembled in this Section, a large number of whom I know have practiced midwifery for many years. There is still, I hold, enough to be learned concerning parturition to engage the attention of even the most accomplished student. I shall confine myself entirely to the management of natural, uncomplicated labor, as my time is limited, and I deem this branch of midwifery of the most practical importance. Meddlesome midwifery is, of course, to be deprecated, and the suggestions I am about to make can only apply to what are termed cases of lingering labor.

There are three stages or conditions in which labor may become lingering, and to deal with these states successfully, different procedures must be made use of. First, labor may be lingering when the head is delayed at the brim of the pelvis; second, when the os has dilated to some extent, and the head has descended into the vagina; and thirdly, when it has reached the vulva and impinges on the perinæum. It is usual to describe lingering labors as occurring in the first and second stages, but I think the division I have adopted will serve better for the elucidation of the subject.

When labor is tardy in the beginning, the os dilating very slowly, the pains feeble and irregular, and the head high up, means may be carefully employed to hasten its progress; but if the woman is cheerful and hopeful, interference may be delayed. This delay must not be suffered to extend, as Churchill and others have recommended, for twelve or sixteen hours, for, if the woman's powers, are allowed to become exhausted in the first stage, instrumental interference becomes a necessity in the second. In the condition described the os is frequently dilatable, but the membranes do not come down to act as a dilating wedge. In these cases it is good practice to detach the membranes around the cervix, and Brown, of Vienna. recommends the introduction of an elastic catheter between the chorion and the walls of the uterus for this purpose; but this can be much better effected, in my judgment, by the cautious use of the finger. After the membranes around the cervix are detached in this way, if gentle pressure is used around the whole margin of the os with the soft part of the finger, gently stretching it, the bag of waters will commence to project, the os will gradually dilate, and the pains become effectual. I have seen many cases of labor in this stage expedited by this plan. In some instances the membranes rupture prematurely, and the head becomes the dilating force. These cases are usually very painful, but they can be greatly hastened if the finger be swept cautiously around the os at each pain. In cases of tardy dilation of the os, due to rigidity, in which the woman suffers acute pain, the administration of opium is most beneficial, and should always be resorted to. A hypodermic injection of ten drops of Majendie's solution, or thirty or forty drops of McMunn's elixir, given internally, acts like a charm. Ineffectual contractions do injury to the woman, and when you cannot advance labor you had better arrest it. I do not think the administration of chloroform is wise at this stage.

There is occasionally a form of unequal, spasmodic contraction, well described by Velpeau, which is sometimes confined to the fundus, sometimes to one of the angles, or to a portion of the anterior or posterior wall, or one of the sides of the uterus. The pains are very acute, but merely exhaust the woman's strength, and, unless an anodyne is administered, labor will be prolonged indefinitely. Belladonna has been used and much praised as a remedy in this condition, but in my hands it has proved utterly useless. Brown's colpeurynter introduced into the vagina, and fully distended with hot water by means of Davidson's syringe, is beyond doubt, the speediest and best means to produce relaxation of all the parts and hasten the labor. It is astonishing how rapidly the os will dilate by the means of this instrument. I have seen by its use in cases of eclampsia, apoplexy, hemorrhage, etc., the os sufficiently dilated in a few hours to admit of the application of the forceps, and a rapid termination of the labor. My experience in cases of this kind h*s taught me its usefulness in normal labor. The colpeurynter may be emptied and re-filled every half hour until dilation is effected.

There is a condition of the uterus which retards labor and gives rise to trouble. The os is dilated to some extent, but the fibers of the cervix above it contract, forming a rim or band. Barnes' hydrostatic bags can be used with great advantage to relax the contracted cervix and allow the descent of the head; but in ordinary cases of contraction I think the colpeurynter serves a better purpose.

The long forceps are frequently resorted to when the head remains for a length of time at the brim of the pelvis, but their application will seldom be necessary if the means I have indicated be cautiously and judiciously employed. I have not found it necessary to perform what is termed the high operation more than three or fonr times in a midwifery practice of more than thirty-seven years. In addition to the measures I have suggested at this stage the patient must be kept out of bed in a vertical position, and not suffered to bear down or make any muscular effort. There is no doubt that the erect posture is the best before the head descends into the lower strait. The conservation of the woman's powers, too, at this period is of the greatest importance.

There is usually no danger to the life of the mother in the first stage of labor as long as the mebranes remain intact, and, on this account, it may be argued that interference is not necessary; but this view I hold is both inhuman and unscientific. Inhuman because it condemns the woman to unnecessary suffering; and unscientific because it leaves agencies unused and powers unaided, which, if employed, would shorten a painful and vital process.

In what I term the second stage, that is when the head has descended into the pelvic cavity, there are two conditions of lingering labor. In the first, though the os may be pretty well dilated, the labor is retarded by the firmness, dryness and want of distensibility of the vagina. Free injections of hot water are useful at this time, and if the membranes be intact it is good practice to rupture them. When the vagina is extremely dry and hot, after the use of the hot water douche, the introduction of a large cotton tampon saturated with glycerine and lard serves a good purpose in softening and dilating the parts. I would add, that if inertia exists at this point, the administration of a drachm or two of the extract of ergot aids the other measures most effectively. But if the arrest of the head is due to occipital-posterior positions it is unsafe and unscientific to administer ergot—the forceps is the only alternative. In these instances there is very little amniotic fluid and no bag of waters is found, indeed I have seen many cases which might absolutely be termed dry labors. After the rupture of the membranes, if strong external compression be used and the os gently stimulated and stretched by the pulpy part of the finger, the pains will be prolonged, the voluntary powers of the woman excited and strengthened, and the labor progress to a speedy close.

The second lingering condition in this stage is when the head is very low down in the pelvis; the os dilated to the size of a half dollar, and found far back toward the sacrum, the head of the child being curved as with a cap by the thinned neck of the uterus. This is a most painful state, and calls loudly for assistance. The membranes, if not ruptured, must be punctured immediately, the os stretched and drawn forward toward the pubis, and strong external pressure used during each pain. Ergot is not generally necessary in this condition, but if the pains are ineffectual, its administration is most beneficial; the labor is accelerated; the woman's voluntary powers are evoked; pain follows pain, and the case has a rapid and happy termination. One of the most trying features in these cases is the intense pain in the region of the sacrum. This can be greatly alleviated by the application to the spine of an embrocation of chloroform and oil of peppermint.

Since writing the foregoing portion of my paper, I assisted in a case, the results of which prove the practicalness of the suggestions I have made.

On the morning of the 28th of May, I was called in by Dr. Ashby, Professor of Obstetrics in the Woman's Medical College of Maryland, to assist him in a case of eclampsia. The doctor had been all night with the woman; had bled her, given hypodermic injections of morphine, and used chloroform very liberally. There was no external evidence of actual labor, but the os was slightly dilated. I ruptured the membranes, distended and stretched the os, pushed down the child by forcible external compression, applied Knight's forceps inside of the uterus, and terminated the labor in forty minutes from the time I reached the house. The child was living.

The most powerful aid in all these cases is forcible external compression. A number of mechanical contrivances have been used to support the abdominal muscles, and secure regular and equal contractions of the uterus, but they are awkward and cumbrous, and do not at all compare in usefulness with the intelligent human hand.

The third and last stage of lingering labor is where the head has descended to the perinæum and owing to inertia of the uterus, or exhaustion of the woman's vital powers, or the rigidity of the muscles or the perinæum, the labor is indefinitely arrested. Hamilton reports a case in which the perinæum was supported in this condition for one hundred and twelve hours. Ergot may be used at this point combined with external compression, but if delivery does not take place speedily the forceps should be applied. Beattie's straight Dublin forceps is the best, being light and easy of application. These are simple tractors and can do no harm. I have observed that if we fail in manipulations with the forceps the labor appears to be arrested and the woman's voluntary powers cease to act, consequently unless one feels convinced that the case will be terminated speedily by instrumental interference, it is better not to attempt it. I have frequently endeavored to extricate the head by passing two fingers into the rectum, but have failed in this maneuver for the reason that the force necessary to be employed is likely to injure the soft parts. The proper management of the perinæum is very important. I have been practicing for years a form of attenuation from the very moment that the head commences to impinge upon the outlet, and I believe that I have greatly assisted the efforts of the women. If the head is still within the uterus at this point, it is good practice to make a sweep with the finger and push the os over the occiput. I generally deliver the patient on the left side, as that position is better for the touch and use of the hand, but sometimes I have thought I have found good results from placing the woman on her back and allowing her to have a few pains in that posture. I am confirmed in this opinion by the experience of a case to which I was called the morning before I left home by Dr. George B. Reynolds, of Baltimore. The head had remained at the outlet for more than three hours without making the slightest progress, when the doctor fortunately changed the position of the patient, and the labor was quickly terminated.

In conclusion, I would state that the great advantage of the procedures briefly suggested in this paper is, that should they fail, they do not interfere with the after-use of the forceps, but rather prepare the way for their easy application. Moreover, I hold that, if properly employed, they prevent those two *bete noire* of modern obstetrical literature, lacerations of the os and perinæum. In addition to this, I believe that post-partum hæmorrhage, that worst complication of midwifery, may also be averted, for it is the weary, out-worked uterus that floods, not the fresh and vigorous organ.

In making these suggestions, I do not wish to be understood as recommending an imitation of the *lesser labours* of the French, where the accoucheur, with rolled-up sleeves, presents himself in front of the patient, and with a great flurry and show of manipulation leads the bystanders to believe that he himself is doing the parturient work—but a scientific employment of measures which experience has proved to be both rational and useful in the furtherance of the greatest physiological process known to mankind. —*Jour. Amer. Med. Association.*

